# Cardiac Adverse Events With Remdesivir in COVID-19 Infection

**DOI:** 10.7759/cureus.11132

**Published:** 2020-10-24

**Authors:** Anupam K Gupta, Barbara M Parker, Vikash Priyadarshi, John Parker

**Affiliations:** 1 Surgery, University of Miami Hospital, Miami, USA; 2 Clinical Pharmacy, AdventHealth Orlando, Orlando, USA; 3 Medicine, Rockledge Regional Medical Center, Rockledge, USA; 4 Obstetrics and Gynaecology, AdventHealth Altamonte, Altamonte Springs, USA

**Keywords:** remdesivir, qtc prolongation, bradycardia

## Abstract

Since December 2019, coronavirus has gradually progressed to a pandemic with no efficacious treatment. Remdesivir is an antiviral medication and inhibitor of viral RNA dependent RNA polymerase with inhibitory action against SARS-CoV virus. Two patients diagnosed with coronavirus infection with worsening respiratory status were initiated with multimodality therapy with antibiotics, steroids and remdesivir. After initiation of remdesivir, the patients’ developed bradycardia, with one of the two also showing signs of worsening QT interval. This reverted upon stopping remdesvir therapy. The prevalence of bradycardia with prolonged QT interval is not well-known yet with this medication.

## Introduction

Remdesivir is an antiviral prodrug developed to treat infections caused by the Ebola virus, Coronavirus’ (SARS-CoV, MERS-CoV), and Nipah virus, respiratory syncytial virus, and Hendra virus [1-3]. Remdesivir was recently approved for compassionate use intravenously for COVID-19 patients [1,2,4,5]. It functions as an adenosine analog that introduces itself into viral RNA, leading to premature chain termination and viral replication inhibition [6,7]. The most common adverse effects of remdesivir are increased hepatic enzymes, diarrhea, anemia, rash, renal impairment, and hypotension [1,2,8,9]. Elevated alanine aminotransferase (ALT) and aspartate aminotransferase (AST) have been shown to be reversible after discontinuation of remdesivir per studies [8-10]. The purpose of our case reports is to highlight two cases who developed adverse drug reactions (ADRs), both with bradycardia, and one with QTc prolongation with T-wave abnormality during remdesivir treatment, which resolved following early discontinuation of the therapy.

## Case presentation

Case 1

A 26 year old African American female presented with cough, chills, nausea, decreased appetite, loose stools, and dry cough for one week. Upon initial evaluation, she had a temperature of 100.3 °F, heart rate of 83, and blood pressure of 120/68 mmHg. A non-rebreather facemask was used to maintain her saturations over 94%. Her medical comorbidities included obesity with a body mass index greater than 35. The nasal polymerase chain reaction was positive for SARS-CoV-2. On admission, her blood work revealed an elevated lactate dehydrogenase (LDH) of 441 IU/L (normal reference: 98-230 IU/L), an elevated c-reactive protein (CRP) of 237 mg/L (normal reference: 0-9mg/L), an elevated procalcitonin of 0.61 ng/mL (normal reference: 0-0.08ng/mL) and an increased d-dimer of 0.54 ug/ml (normal reference: 0.27-0.5). Her liver and renal function tests were within normal limits. Computed tomography of the chest showed extensive bilateral consolidative changes. Due to her worsening respiratory status, she was initiated on a multimodal therapy of the antibiotics ceftriaxone and azithromycin, the steroid methylprednisolone, convalescent plasma and remdesivir.

**Figure 1:**
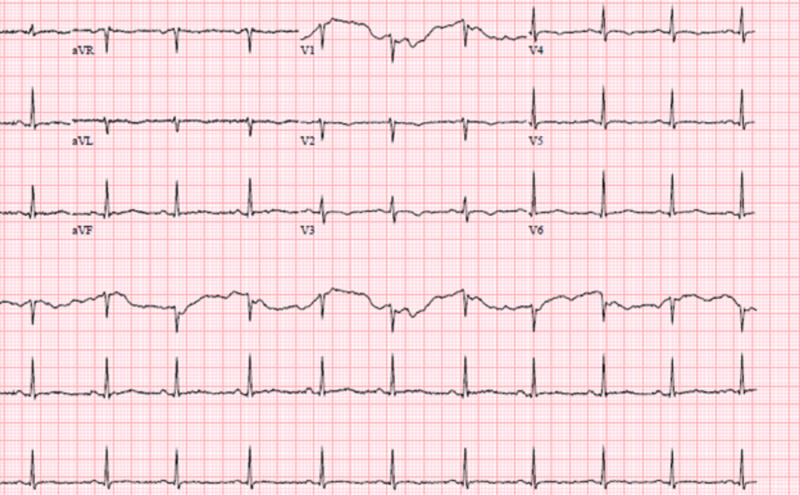
EKG showing normal sinus rhythm with ventricular rate 77bpm and normal QT/QTc interval 388/439 ms

**Figure 2:**
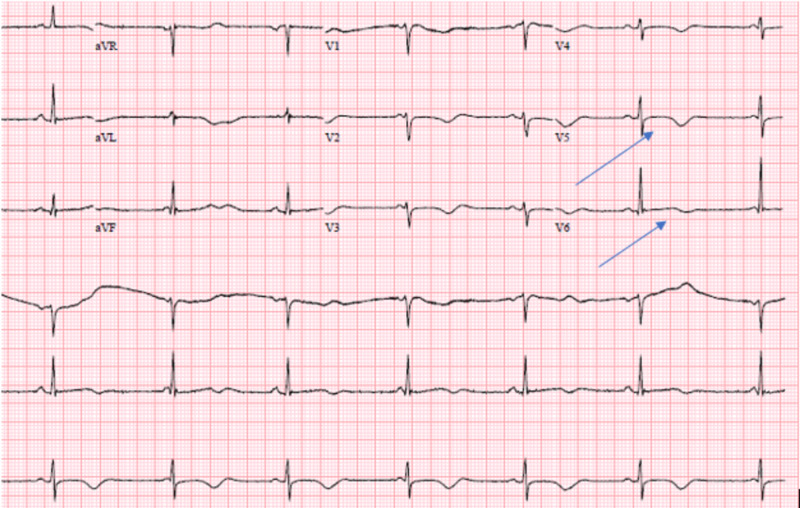
EKG on the third day of remdesivir treatment. Marked sinus bradycardia with ventricular rate of 44 bpm, nonspecific T wave abnormality, and prolonged QT/QTc interval 628/555 ms

**Figure 3:**
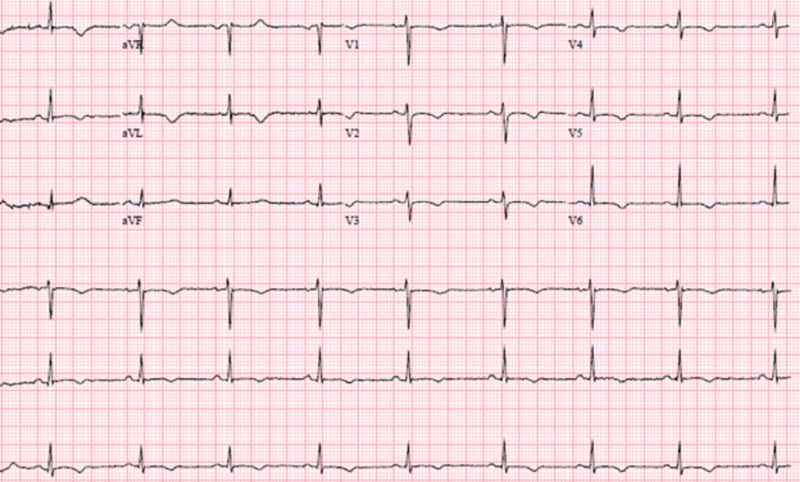
EKG two days post-remdesivir discontinuation showing sinus rhythm, with ventricular rate of 64 bpm, and normalizing QT/QTc of 448/448 ms

**Figure 4:**
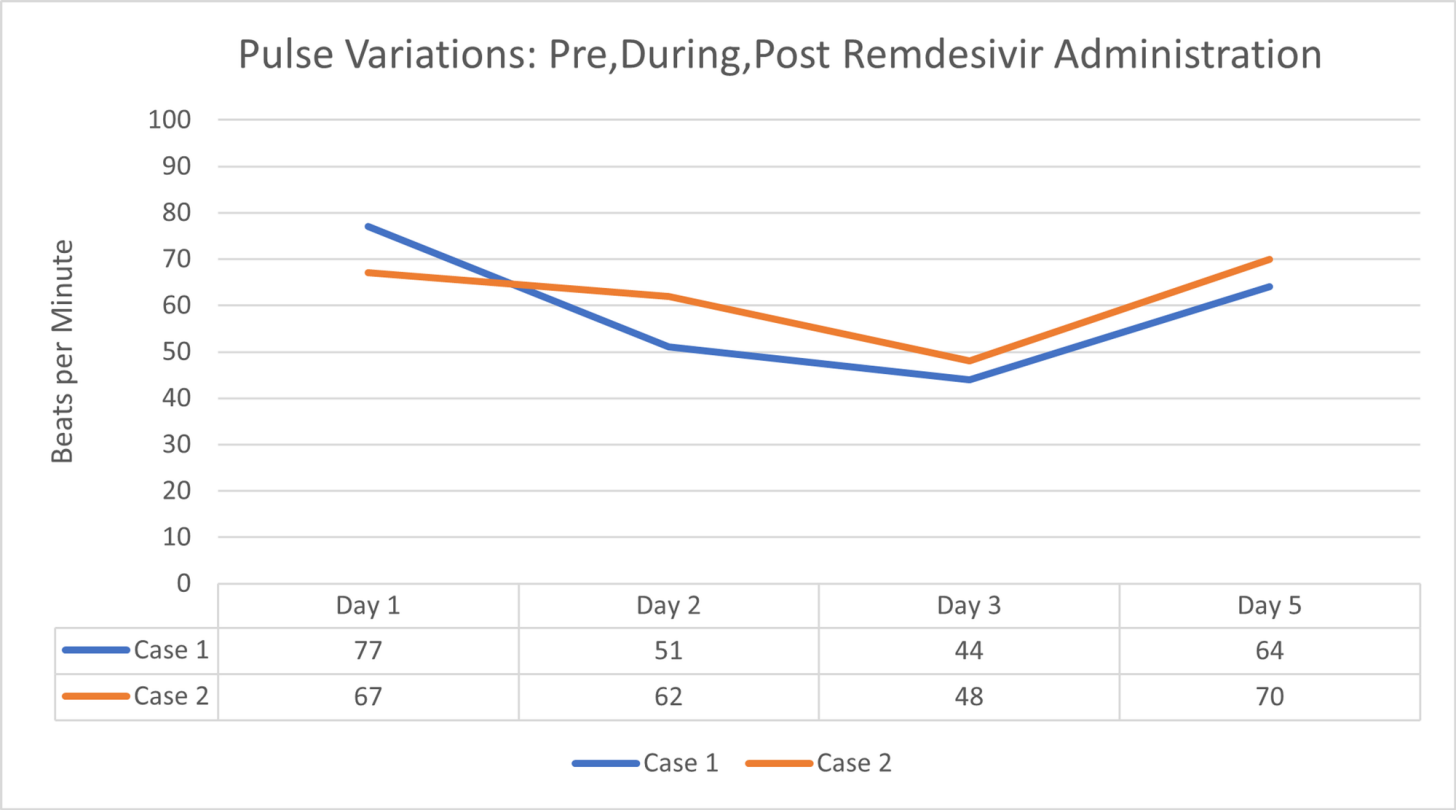
Changes in heart rate (bpm) from day one to day three (time of discontinuation) of a five day course of remdesivir treatment for patient cases one and two

At baseline she was in normal sinus rhythm with a heart rate of 80 to 100 bpm and a QTc interval of 439 ms. Her baseline EKG showed no rhythm abnormalities (Figure 1). After her third dose of a five-day treatment course of remdesivir, she was in sinus bradycardia with her heart rate dropping to 40-50 beats per minute, prolonged QTc interval of 555 ms, and T wave abnormality (Figure 2). The remdesivir treatment was discontinued and the patient’s heart rate returned to baseline with her QT interval stabilizing to 448ms in three days (Figure 3,4).

Case 2

A 77-year-old Caucasian female presented with complaints of weakness, headache, and loose stools for a week. She was febrile with a maximum temperature of 100.1 °F; blood pressure of 115/60 mmHg, and in sinus rhythm with a heart rate 67 bpm. Blood work was positive for elevated creatinine to 1.4, mild transaminitis with AST of 104, ALT of 64, C-reactive protein of 245, d-dimer of 2.13, lactate dehydrogenase of 263 international units/L, and procalcitonin of 0.97 ng/mL. The patient's chest x-ray showed atypical infiltrates, and her nasal polymerase chain reaction for coronavirus was positive. The patient was also initiated on multimodality therapy with the antibiotics, ceftriaxone and azithromycin, steroid methylprednisolone, and remdesivir. On day three after remdesivir administration, the patient developed sinus bradycardia with a heart rate dropping from 80 bpm to 48 bpm. Consequently, remdesivir was discontinued, and the patient’s heart rate returned to baseline on subsequent days (Figure 4).

## Discussion

Remdesivir is a broad-spectrum antiviral drug with antiviral effect on coronaviruses, including MERS-CoV and SARS-CoV [1-4,10-12]. Studies have shown that almost 80% of the genome of SARS-COV-2 is homologous to that of SARS-CoV, and almost all SARS-CoV-2 proteins are homologous to SARS-CoV proteins, supporting its use for both coronavirus types [2,3,5-7]. Treatment courses of remdesivir for COVID-19 include a loading dose of 200mg intravenously followed by a 100mg daily dose for 5 to 10 days [8].

Adverse effects are common with remdesivir, but few studies exist that focus on remdesivir and its effects on the cardiovascular system [1,2,9,10,12,13]. Out of a study of 53 patients receiving remdesivir, 32 patients (60%) experienced adverse events during follow-up [5]. These adverse events were increased hepatic enzymes, diarrhea, rash, renal impairment, and hypotension [5]. Adverse events were more common for those on mechanical ventilation compared to those that were not [5]. Bradycardia or EKG changes were mentioned in this cohort study which would pertain to our findings in this case report and neither of the patients in our study progressed to mechanical ventilation [5]. One study evaluating cardiovascular safety reports a decreased potassium level in patients with COVID-19 which may be contributing to a prolongation of the QT interval on EKG [10]. However, our 26 year old patient who experienced a QT interval prolongation had a potassium within the normal range, indicating potassium is less likely to have played a significant role. It is important to note that this patient was on azithromycin the day prior to discontinuation of remdesivir which is well known to prolong the QT interval. It is possible that it contributed to the prolongation of the QT interval in this patient despite having been discontinued shortly before remdesivir initiation. Remdesivir itself has been mentioned in its own right to have an effect on the QT interval, but more studies are needed to support a causality [8,10,12]. Cardiac disturbances in COVID-19 patients receiving remdesivir seem to be primarily due to bradycardia or hypotension in the studies found and less commonly being due to QT prolongation [4,5,10,14,15].

Many pitfalls still exist with understanding COVID-19 and the use of remdesivir. Optimum time of administration in relation of the benefit of remdesivir for COVID-19 has yet to be clear, although initiation is generally considered once oxygen saturations drop below 94% [1,2,8,14]. Both of our patients developed sinus bradycardia within 20-40 minutes of infusion time (at drug peaks), and typically doses are infused over a full hour to completion [8]. The coronavirus’ also have a favorable profile toward the development of remdesivir resistance, which may explain why 5 days of therapy is commonly utilized over 10 days [7]. Strikingly, some studies have shown the placebo group was just as likely if not more likely to have adverse events compared to those who received remdesivir, which suggests a link between cardiac changes and COVID-19 illness; this supports the need for better powered and more high quality studies (randomized controlled trials) [11-13,15].

## Conclusions

Some SARS-CoV-2 patients on remdesivir develop sinus bradycardia and a prolonged QT interval. Appropriate caution and continuous EKG monitoring should be utilized in all patients participating in ongoing trials for COVID-19 as the safety of remdesivir remains largely uncertain. Even closer surveillance for patients with pre-existing heart disease is warranted when using remdesivir. There remains the need for more high quality evidence from randomized controlled trials presently underway. Attention to additive cardiovascular adverse effects from other drug classes remains crucial to ensure positive patient outcomes and to minimize risk of potential fatal arrhythmias or cardiac arrest.
